# Anti‐angiogenic effects of the blue‐green alga *Arthrospira platensis* on pancreatic cancer

**DOI:** 10.1111/jcmm.14922

**Published:** 2020-01-19

**Authors:** Ivana Marková, Renata Koníčková, Kateřina Vaňková, Martin Leníček, Michal Kolář, Hynek Strnad, Miluše Hradilová, Jana Šáchová, Jan Rasl, Zuzana Klímová, Tomáš Vomastek, Ivana Němečková, Petr Nachtigal, Libor Vítek

**Affiliations:** ^1^ Institute of Medical Biochemistry and Laboratory Diagnostics Faculty General Hospital and 1st Faculty of Medicine Charles University Prague Czech Republic; ^2^ Institute of Molecular Genetics of the Czech Academy of Sciences Prague Czech Republic; ^3^ Department of Informatics and Chemistry Faculty of Chemical Technology University of Chemistry and Technology Prague Czech Republic; ^4^ Institute of Microbiology of the Czech Academy of Sciences Prague Czech Republic; ^5^ Department of Cell Biology Faculty of Science Charles University Prague Czech Republic; ^6^ Department of Biological and Medical Sciences Faculty of Pharmacy in Hradec Kralove Charles University Hradec Králové Czech Republic; ^7^ 4th Department of Internal Medicine Faculty General Hospital and 1st Faculty of Medicine Charles University Prague Czech Republic

**Keywords:** angiogenesis, anticancer effects, *Arthrospira platensis*, carcinogenesis, pancreatic cancer

## Abstract

*Arthrospira platensis*, a blue‐green alga, is a popular nutraceutical substance having potent antioxidant properties with potential anti‐carcinogenic activities. The aim of our study was to assess the possible anti‐angiogenic effects of *A platensis* in an experimental model of pancreatic cancer. The effects of an *A platensis* extract were investigated on human pancreatic cancer cells (PA‐TU‐8902) and immortalized endothelial‐like cells (Ea.hy926). PA‐TU‐8902 pancreatic tumours xenografted to athymic mice were also examined. In vitro migration and invasiveness assays were performed on the tested cells. Multiple angiogenic factors and signalling pathways were analysed in the epithelial, endothelial and cancer cells, and tumour tissue. The *A platensis* extract exerted inhibitory effects on both migration and invasion of pancreatic cancer as well as endothelial‐like cells. Tumours of mice treated with *A platensis* exhibited much lesser degrees of vascularization as measured by CD31 immunostaining (*P* = .004). Surprisingly, the VEGF‐A mRNA and protein expressions were up‐regulated in pancreatic cancer cells. *A platensis* inhibited ERK activation upstream of Raf and suppressed the expression of ERK‐regulated proteins. Treatment of pancreatic cancer with *A platensis* was associated with suppressive effects on migration and invasiveness with various anti‐angiogenic features, which might account for the anticancer effects of this blue‐green alga.

## 1 INTRODUCTION

Nutritional factors contribute substantially to the pathogenesis of cancer, particularly in Western‐style diets that are rich in saturated fats and lacking in protective nutrients. Among other factors, food‐borne compounds capable of scavenging reactive oxygen species are believed to be especially important in the prevention of oxidative stress‐mediated diseases.^1^ Besides being found in fruits and vegetables, these compounds are also present in many edible algae, which while quite popular in some Asian countries are rarely used in Western‐style diets.^2^

*Arthrospira platensis* (*Spirulina platensis*) is a freshwater blue‐green alga known for its remarkable biological activities. For example, the strong anti‐infective effects of *Spirulina* algae extracts against the human immunodeficiency virus have been demonstrated in in vitro studies, and regions with a high consumption of these nutrients (such as Chad or Eastern Asia) have a far smaller prevalence of AIDS compared to neighbouring countries, known to not consume these nutrients.^3^ Algae consumption might also be associated with a decreased prevalence of cancer, as demonstrated in experimental,^4^ as well as some scarce epidemiological studies.^5^ These algae contain a large number of potentially active substances including iodine, selenium, folate, carotenoids, chlorophyll, the digestible algae polysaccharides alginic acid and fucoidin, and n‐3 polyunsaturated fatty acids^2^—any of which might contribute to the antioxidant and antiproliferative biological effects.^6^, ^7^, ^8^, ^9^ Certain algae, including *A platensis*, also contain other potentially active substances that belong to a family of phycobilins (in particular phycocyanobilin), which are linear tetrapyrrolic compounds related to human bile pigments, known to have strong antioxidant as well as antiproliferative properties.^10^ Phycocyanobilin linked to a specific protein moiety forms a molecule called C‐phycocyanin exerting potent biological effects, including anticancer activities.^11^ However, molecular mechanisms accounting for these antiproliferative effects are largely unknown.

We recently reported the potent suppressive effects of *A platensis* on the growth and proliferation of experimental pancreatic cancer.^4^ The RAS‐regulated RAF‐MEK1/2‐ERK1/2 pathway, with possible impacts on angiogenesis in the cancer tissue,^12^, ^13^ is dysfunctional in pancreatic cancer.^14^, ^15^ In fact, anti‐angiogenic therapeutic approach targeting the vascular endothelial growth factor (VEGF) or the epidermal growth factor receptor (EGFR) signalling has become a promising strategy in the treatment of pancreatic cancer^16^, ^17^ with the aim to modulate protein kinase B (AKT) and extracellular signal‐regulated kinase (ERK) (pAKT and p‐ERK) pathways dysregulated in these cancers.^18^

Thus, the aim of this current study was to evaluate the possible anti‐angiogenic effects of *A platensis* to account for the antiproliferative effects of this alga.

## 2 MATERIALS AND METHODS

### 2.1 Materials

The *A platensis* was purchased from Martin Bauer GmbH (Vestenbergsgreuth, Germany). The water extract of both *A platensis* and phycocyanobilin was prepared as has been previously described elsewhere.^4^ The cell culture media and non‐essential amino acids (NEAAs) were obtained from Sigma‐Aldrich, and the other cell culture components were from Biosera (Nuaille, France). The serine/threonine phosphatase and protease inhibitor cocktails were purchased from either Sigma‐Aldrich or Serva. The Geltrex™ LDEV‐Free Reduced Growth Factor Basement Membrane Matrix was purchased from Thermo Fisher Scientific. The recombinant growth factors and inhibitors were procured as follows: rVEGF, rEGF (epidermal growth factor), rAREG (amphiregulin, autocrine mitogen related to EGF), rHGF/SF (hepatocyte growth factor/scatter factor), PD 0325901 (all from Sigma‐Aldrich), erlotinib (Cell Signaling Technology), vatalanib and axitinib (Selleck Chemicals) and bevacizumab (LGM Pharma). Unless otherwise specified, all other common chemicals were from Sigma‐Aldrich.

### 2.2 Cell lines

The human pancreatic ductal adenocarcinoma PA‐TU‐8902 cells (DSMZ), MIA PaCa‐2, PANC‐1 and BxPC‐3 cells (ATCC), immortalized human endothelial‐like cells (EA.hy926; ATCC), and MDCK‐ΔRaf‐1:ER cells, stably expressing conditionally active Raf,^19^ were used for the in vitro experiments. The cells were cultured in a humidified atmosphere (containing 5% CO_2_ at 37°C) in a DMEM supplemented with 10% foetal bovine serum (FBS), 1% penicillin/streptomycin, 1% NEAAs, 1% glutamine and in 2% HAT supplement (EA.hy926). For some experimental studies, a low‐serum medium, with 0.5% FBS, was used. To activate the ERK pathway, the MDCK‐ΔRaf‐1:ER cells were cultured in a DMEM with 10% FBS and treated with either 1 μmol/L 4‐hydroxytamoxifen (4HT) or 100 ng/mL rHGF/SF. The PA‐TU‐8902 and EA.hy926 cell lines were authenticated at ATCC by STR profiling before distribution and were also re‐authenticated at the end of the study (Generi Biotech).

### 2.3 Tumour tissue from in vivo experiments

Pancreatic cancer xenografts (PA‐TU‐8902 cells) from our previous study on mice treated with biologically relevant doses of *A platensis* extract^4^ were used for the Western blot, immunohistochemical staining, angiogenic proteome and mRNA expression analyses. In these studies, tumour sizes were significantly smaller as early as the third day after initiation of the *A platensis* extract treatment reaching only 40% of the size of untreated animals in 2 weeks of treatment.^4^ The mice were killed after 2 weeks of intragastric administration of a water suspension of freeze‐dried *A platensis* (0.5 g/kg once daily); after, the tumour tissue specimens were sampled and stored at −80°C until analysed.

All aspects of the animal studies and all protocols met the accepted criteria for the care and experimental use of laboratory animals, and were approved by the Animal Research Committee of the 1st Faculty of Medicine, Charles University, Prague (under registration no. 356/10). All procedures were performed under *lege artis* conditions, and all efforts were made to minimize animal suffering.

### 2.4 Cell viability assays

The effect of growth factors (VEGF; EGF; AREG at concentrations of 0.1, 1, 10, 50, 100 μg/L) on the viability of PA‐TU‐8902 pancreatic cancer and EA.hy926 endothelial‐like cells was measured by a MTT viability assay.

### 2.5 Tube‐like formation assay

Immortalized EA.hy926 cells that retain several endothelial characteristics were used to determine the effect of *A platensis* on angiogenesis. These EA.hy926 endothelial‐like cells (2.5 × 10^4^ cells per well) pretreated with a water extract of *A platensis* (0.3 g/L) for 24 hours were seeded in a 96‐well plate covered with a Geltrex™ basement membrane matrix, with reduced growth factors in DMEM supplemented with 0.5% serum in either the presence or absence of a water extract of *A platensis*. The formation of tube‐like structures was inspected, and photographs were taken after 24 hours using an Olympus TL4 microscope (Shinjuku, Tokyo, Japan). The images were analysed by the Angiogenesis Analyzer tool in ImageJ software (NIH), and the total length of the tube‐like structures was calculated as the length of the tubes relative to the control cells.

### 2.6 Wound‐healing assay

PA‐TU‐8902 or EA.hy926 cells were seeded into 12‐well plates at a density of 2 × 10^5^ cells/well in complete DMEM and cultured to 100% confluence. The cells were starved in a low‐serum medium 6 hours prior to the experiment. The confluent cell monolayer was then scratched with a pipette tip and washed three times with Hank's solution to remove cell debris. The cells were incubated at 37°C for 24 hours in a low‐serum medium (DMEM supplemented with 0.5% serum) with a water extract of *A platensis.* To quantify cell migration, images of the wound were taken 0 and 24 hours after being scratched (Olympus TL4 microscope, Tokyo, Japan), and the images were then processed with ImageJ software. The rate of cell migration was then calculated as the area filled by cells migrating into the denuded area (in square pixels) and plotted as the average from at least 3 independent experiments in quadruplicate.

### 2.7 Cell migration and invasion assay

Cultrex^®^ in vitro angiogenesis and endothelial cell invasion assays (Trevigen) were used for the cell migration and invasiveness studies. The EA.hy926 cells were first starved in a low‐serum medium for 6 hours and then pretreated with a low‐serum medium for the next 6 hours with the water extract of *A platensis* (0.3 g/L). The cells were harvested, re‐suspended in a serum‐free medium with the water extract of *A platensis* (0.3 g/L) and then seeded onto the upper insert of a 96‐well plate (2 × 10^4^ cells per well) prepared according to the manufacturer's instructions. The membranes of the wells on the upper insert plate were covered with the basement extracellular membrane extract depending on whether being tested for invasion or migration. Cells that penetrated across the membrane were detected after 24 hours by measurement of the fluorescence signal (excitation 485 nm, emission 520 nm) after calcein‐AM internalization using a microplate reader (Infinite^®^ 200, Tecan).

### 2.8 Western blot analyses

Samples were separated by SDS‐PAGE, blotted onto a nitrocellulose or PVDF membrane, blocked for 1 hours with 5% non‐fat dry milk in TBS‐Tween (0.05%‐0.1%) or in PBS‐Tween and then probed with the corresponding primary and secondary antibodies (Table S1). The chemiluminescent signal of horseradish peroxidase (HRP)–conjugated antibodies was detected on film (CD31, VEGF‐A) or digitally processed by a Fusion Fx7 detection system (Vilber Lourmat, Collégien, France). For determination of ERK and AKT activation, PA‐TU‐8902 and MDCK cells grown at 50% confluency were lysed in a RIPA buffer supplemented with phosphatases and proteases inhibitors as described above. The signals of fluorescently labelled secondary antibodies (for ERK, AKT and p120RasGap detection) were visualized by use of an Odyssey Infrared Imaging System (LI‐COR Biosciences) The HRP chemiluminescent signal was detected by either exposure to blue autoradiography films or collected by G:BOX Chemi XRQ gel system (Syngene, UK). Fluorescent or chemiluminescent signals were normalized to p120RasGap expression using Quantity One software (Bio‐Rad Laboratories). Equal loading of the proteins onto the gel was confirmed by the immunodetection of β‐actin signal for CD31, VEGF‐A, p120RasGap, ERK1/2, p90RSK (phospho‐ERK, N‐cadherin, FRA1) or Ponceau staining (EGFR)—as indicated in the figure legends.

For CD31/VEGF‐A, using the Western blot analyses from pancreatic cancer xenografts, the excised tissue was homogenized in a RIPA lysis buffer. The chemiluminescent process and quantification of the immunoreactive bands on the exposed films were carried out as have previously been described.^20^

For the EGF receptor (EGFR) protein expression, PA‐TU‐8902 cells were incubated for 24 hours in a medium with or without *A platensis.* Ten minutes before cell lysis in RIPA buffer, supplemented with phosphatases and proteases inhibitors, rEGF or rAREG was added at a final concentration of 50 ng/mL. Quantification and statistical analyses of the Western blots were performed from at least three independent experiments run in triplicate.

### 2.9 Immunohistochemistry

Cryosections of tumour tissue were fixed in acetone at −20°C for 30 minutes. Slides were blocked with 10% normal goat serum (Sigma‐Aldrich) in PBS (pH 7.4) for 30 minutes. For the detection of CD31, the slides were thereafter incubated with primary antibody for 1 hour at room temperature (rabbit anti‐CD31, Santa Cruz Biotechnology; dilution 1:100 in BSA) and were developed with goat anti‐rabbit IgG conjugated to peroxidase‐labelled polymer EnVision for 30 minutes at room temperature. For the detection of VEGF‐A, the slides were first incubated with anti‐avidin and anti‐biotin solutions (Vector Laboratories). Afterwards, they were incubated with primary antibody overnight at 4°C (rabbit anti‐VEGF‐A, Santa Cruz Biotechnology; dilution 1:50 in BSA) and then developed with biotin‐conjugated goat anti‐rabbit Ig (dilution 1:400 in BSA) (Vector Laboratories) for 30 minutes at room temperature and subsequently with HRP‐conjugated avidin‐biotin complex (VECTASTAIN Elite ABC Kit, Vector Laboratories) for 30 minutes. Visualization of bound antibodies was performed using diaminobenzidine‐tetrahydrochloride substrate (DAB, Dako). All sections were counterstained by haematoxylin. Photo documentation and image digitizing were performed using Olympus AX 70 (Olympus) with a digital firewire camera Pixelink PL‐A642 (Vitana Corporation) and image analysis software NIS‐Elements, version 5.0 (Laboratory Imaging, Czech Republic).

### 2.10 Quantitative real‐time PCR

Total RNA was extracted using a PerfectPure™ RNA Cell Isolation Kit (5 PRIME GmbH, Hilden, Germany). The cDNA was prepared using a High‐Capacity cDNA Reverse Transcription Kit (Applied Biosystems). The cDNA was mixed with SYBR GREEN master mix (Applied Biosystems), plus with specific primers for genes of interest (Table S2), and then examined by RT‐PCR on a ViiA™ cycler (Applied Biosystems).

### 2.11 VEGF‐A, AREG and EGF protein determination

For measuring the effect of *A platensis* extract on VEGF‐A, AREG and EGF production, PA‐TU‐8902 cell media were collected after 24‐hour incubation, with or without water extract of *A platensis* (0.3 g/L). To determine whether secretion of VEGF‐A persisted even without the continuous presence of *A platensis* (0.3 g/L), the medium of PA‐TU‐8902 cells after 12 hours of incubation with *A platensis* was changed to a medium without *A platensis.* The medium was harvested after the next 12 hours. The effects of different inhibitors of the VEGF signalling pathway (anti‐angiogenic tyrosine kinase inhibitors vatalanib [1 μmol/L] and axitinib [50 nM] as well as phycocyanobilin [125 μmol/L]) were also tested. For these experiments, cells were starved in a low‐serum medium for 24 hours; the medium was then changed to the full medium, with or without a corresponding agent. The medium was taken after a 5‐hour‐long incubation. AREG secretion was also measured for cells exposed to *A platensis* and/or other compounds regulating the VEGFR and EGFR pathway (erlotinib [1 μmol/L ≈ 0.4 mg/L], bevacizumab, a monoclonal antibody against VEGF‐A [25 μg/L], rVEGF‐A/rEGF [50 μg/L]) for 24 hours. All media were centrifuged to eliminate dead cells, and the supernatant was stored at −80°C until analysed.

The concentrations of VEGF‐A, AREG and EGF secreted into the medium of cultured pancreatic cancer cells were detected by ELISA (Human VEGF‐A ELISA Kit, RayBiotech, Inc; Human Amphiregulin ELISA Kit and Human EGF ELISA Kit, both Thermo Fisher Scientific) according to the manufacturer's instructions.

### 2.12 Determination of the angiogenic proteome

The effect of the experimental treatment on angiogenic proteins in human pancreatic cancer xenografts as well as in vitro cultured pancreatic cancer cells (2‐day exposure of PA‐TU‐8902 cells to the *A platensis* extract [0.6 g/L]) was evaluated using a Proteome profiler angiogenesis array (RD System) covering 55 key angiogenic factors, according to the manufacturer's instructions. Briefly, the cell lysate was incubated with a cocktail of biotinylated antibodies, and the final mixture was then incubated with an array membrane spotted with specific capture antibodies. The resulting complex was detected by streptavidin‐HRP with chemiluminescent detection (Fusion Fx7 detection system, Vilber Lourmat).

### 2.13 Statistical analyses

The statistical significance of the differences between variables was evaluated by *t* test or Mann‐Whitney rank‐sum test. Differences between multiple groups were assessed by ANOVA or Kruskal‐Wallis rank‐sum test with post hoc Dunn's test. Depending on their normality, data are presented as the mean ± SD, or the median and IQ range. Differences were considered statistically significant when the *p*‐values were <.05.

For comparison of the effects of *A platensis* in cells stimulated with either HGF/SF or 4HT (Figure 6), data from two independent experiments were analysed, each performed in duplicates. Two‐way ANOVA was performed to assess an additive batch effect between the experiments. Twofold changes in ERK phosphorylation with false discovery rate (FDR) <0.05 were considered statistically significant. Batch‐corrected values were plotted (Figure 6), with the median of the controls set to zero. All analyses were performed in R.

## 3 RESULTS

### 3.1 *Arthrospira platensis* inhibits migration of both pancreatic cancer and endothelial cells

**Figure 1:**
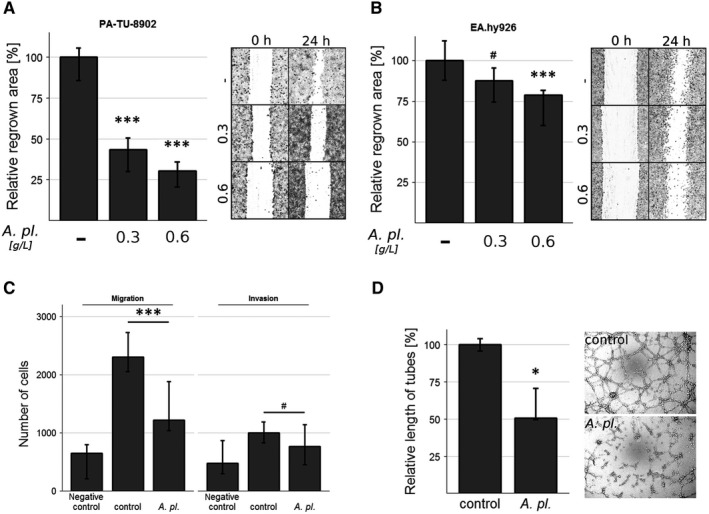
Inhibitory effect of *Arthrospira platensis* extract on wound‐healing performed on both (A) human PA‐TU‐8902 pancreatic cancer and (B) EA.hy926 endothelial‐like cells. Cell migration measured as the capacity of cells to regrow the wound area after 24‐h incubation with or without extract (0.3 or 0.6 g/L). Data expressed as % of control values and represent median ± IQ range. ^p^ *P* = .058 vs control; *P < .05 vs control; ***P < .001 vs control. C, Migration and invasiveness performed on EA.hy926 endothelial‐like cells. Data expressed as the number of cells that migrated in 24 h through porous membrane covered or not by basement extracellular membrane extract layer towards medium containing serum (control, *A pl.* group) or not (negative control group), and represent median ± IQ range. ^p^ *P* = .062 vs control; ***P < .001 vs control. D, Tube‐like formation performed on EA.hy926 endothelial‐like cells. Total tube‐like structure length was measured after 24‐h incubation with or without *A platensis* extract (0.3 g/L); *, *P* < .05, *A pl.*,* Arthrospira platensis*

The migration capacity of cancer cells is an important factor for tumour progression. Thus, we assessed the effect of *A platensis* with regard to this phenomenon. We observed that the *A platensis* water extract (0.3 g/L) significantly suppressed wound healing in a scratch assay performed on PA‐TU‐8902 pancreatic cancer cells (*P* < .001, Figure 1A), as well as (to a lesser extent) also on EA.hy926 endothelial‐like cells (Figure 1B). The potential of *A platensis* extract to inhibit cell migration of EA.hy926 cells was also confirmed in the Boyden chamber transwell migration assay as well as in the invasion assay. Migration was significantly suppressed (by 47%, *P* < .001, Figure 1C); also, the drop in invasiveness of the cells was remarkable and reached borderline significance (*P* = .062, Figure 1C).

### 3.2 *Arthrospira platensis* inhibits angiogenesis in vitro

As tumour progression is dependent upon angiogenesis, we investigated the possible role of *A platensis* on the functional capacity of endothelial cells. Treatment with the *A platensis* extract (0.3 g/L) significantly reduced the total length of the tube‐like structures of EA.hy926 cells (to approximately 50% that of the control cells, *P* < .05, Figure 1D); although, as mentioned above, the viability of the endothelial cells was not compromised.

### 3.3 *Arthrospira platensis* decreases vascularization in human pancreatic cancer xenografts

**Figure 2:**
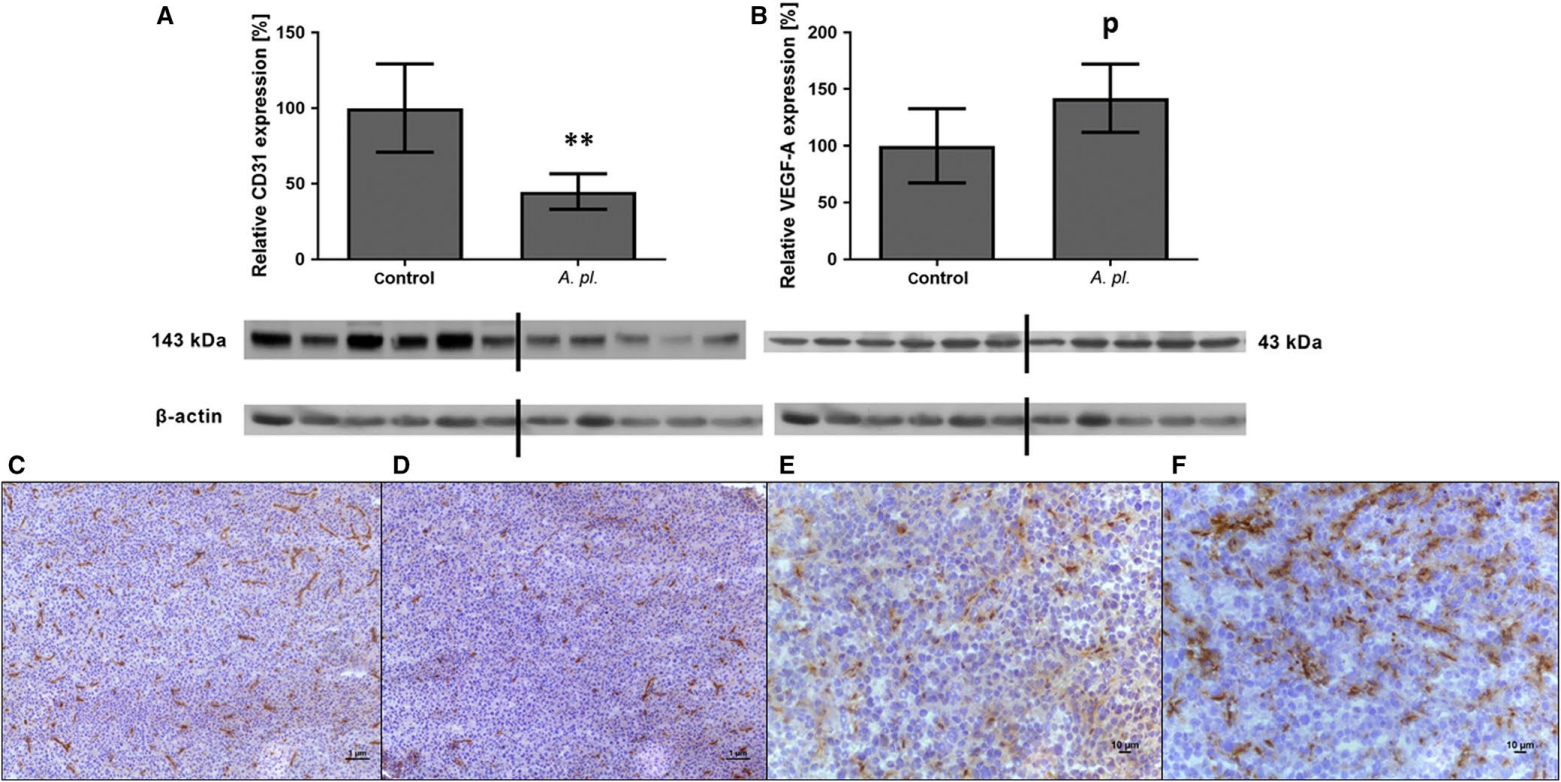
Protein expression of CD31 and VEGF‐A in human pancreatic tumours grown in athymic mice treated orally with *Arthrospira platensis* for 2 wk. Western blot analysis of CD31 (A) and VEGF‐A expression (B), and representative microphotographs of CD31 immunohistochemical staining of control (C) and *A platensis*‐treated mice (D) and VEGF‐A staining of control (E) and *A platensis*‐treated mice (F). Densitometric quantification of immunoreactive bands was performed by recalculation to the β‐actin signal. The positivity for both antibodies is visualized by brown staining and counterstaining by haematoxylin. Bar 1 μm and 10 μm. Data in (A) and (B) represent median ± IQ range, ^p^ *P* = .056 vs control; **P < .01 vs control. The Western blot lanes represent the individual mouse tumour lysates. VEGF‐A, vascular endothelial growth factor A; *A pl.*,* Arthrospira platensis*

Due to the beneficial effects of *A platensis* on angiogenesis observed in the in vitro assays, we focused on an evaluation of the vascularization of tumours, as measured by the expression of endothelial marker CD31.^21^ Indeed, as compared to the controls, the expression of CD31 was more than 2X lower in the tumours of animals treated with *A platensis* (45% of controls, *P* < .01, Figure 2A).

### 3.4 *Arthrospira platensis* stimulates VEGF‐A production in human PA‐TU‐8902 pancreatic cancer cells

Although angiogenesis is a complex process, being regulated and modified by multiple factors, VEGF‐A is considered the key player.^22^ However, despite the significantly lower expression of the CD31 capillary marker in the tumour mass of animals treated with *A platensis* (Figure 2A), the VEGF‐A protein expression was surprisingly higher in tumour tissues, reaching borderline significance (by 48%, *P* = .056, Figure 2B). Immunohistochemical staining of tumours showed stronger CD31 positivity in capillaries of the tumour tissue in control animals when compared to animals treated with *A platensis* (Figure 2C,D). VEGF‐A staining was positive in both capillaries and stromal cells of the tumour in both control and *A platensis‐*treated animals, but the staining was more intensive in animals treated with *A platensis* (Figure 2E,F).

**Figure 3:**
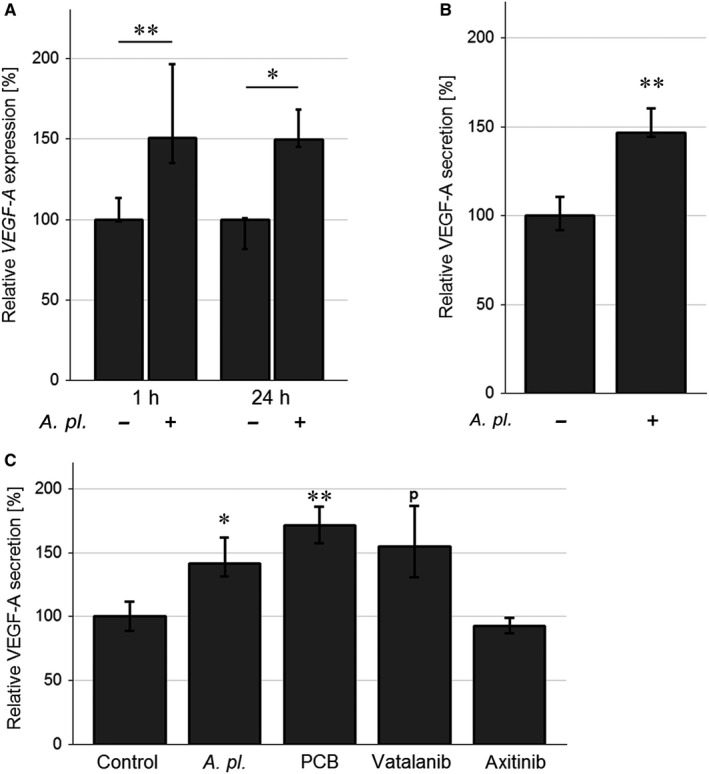
VEGF‐A production by human PA‐TU‐8902 pancreatic cancer cells exposed to *Arthrospira platensis* and various anti‐angiogenic drugs: (A) *VEGF‐A* mRNA expression after 1‐ and 24‐h exposure to *A platensis* extract. B, VEGF‐A protein production upon 24‐h exposure to *A platensis* extract. C, VEGF‐A protein production upon 5‐h exposure to various anti‐angiogenic drugs. The VEGF‐A mRNA level was determined by RT‐qPCR. The concentrations of VEGF‐A secreted into the medium of PA‐TU‐8902 cells were detected by ELISA. The following concentrations of anti‐angiogenic factors were used: *A platensis* extract (0.3 g/L), PCB (125 μmol/L), axitinib (50 nmol/L) and vatalanib (1 μmol/L). Data expressed as % of control values and represent median ± IQ range. ^p^ *P* = .054 vs control; *P < .05 vs control; **P < .01 vs control. *A pl.*, *A platensis*; PCB, phycocyanobilin

These results were confirmed in the in vitro experiment with the PA‐TU‐8902 cells exposed to *A platensis* (0.3 g/L). The *VEGFA* mRNA expression was significantly up‐regulated after both 1 and 24 hours of *A platensis* exposure, by 50% and 49%, respectively (*P* < .05 for both comparisons, Figure 3A). This overexpression was reflected by increased VEGF‐A secretion after 24 hours of exposure to *A platensis* extract (by 47%, *P* < .01, Figure 3B). A similar stimulatory effect on VEGF‐A production after just 5 hours of exposure was not only observed for *A platensis*, but also for phycocyanobilin and vatalanib (a selective VEGF receptor (VEGFR) inhibitor), while no effect was found for axitinib (a less specific tyrosine kinase inhibitor) (Figure 3C).

The effect of *A platensis* extract on VEGF‐A production by PA‐TU‐8902 cells was long‐standing, as increased VEGF‐A production persisted for as long as 12 hours after exchanging the culture media with one not containing *A platensis* extract (data not shown).

To assess whether up‐regulated VEGF‐A production in PA‐TU‐8902 cells was also associated with changes in mRNA expressions of VEGFR, we analysed both VEGFR1 and VEGFR2 mRNA levels on exposure of these cancer cells to the *A platensis* extract. However, no changes in VEGFR1 or VEGFR2 were detected regardless of the exposure status, suggesting that VEGF‐A overproduction by PA‐TU‐8902 cells exposed to *A platensis* extract did not have any positive autocrine effect on VEGFR expression. Similarly, no change in VEGFR1 protein expression was found in the tumour tissue excised from the mice treated *A platensis* extract, as well as in EA.hy926 endothelial‐like cells exposed to *A platensis* extract (data not shown). We also determined whether VEGF‐A overproduced upon exposure to *A platensis* can have paracrine effects on the proliferation of pancreatic cancer cells. To do this, we tested the viability of PA‐TU‐8902 cells exposed to recombinant VEGF‐A (within a concentration range of 10‐100 ng/mL). However, no changes in the cell viability/proliferation status were observed at any of the concentrations used (data not shown).

### 3.5 *Arthrospira platensis* modulates protein expression of numerous angiogenic factors in human pancreatic cancer cells

Based on the overproduction of VEGF‐A, despite the lower vascularization and decreased proliferation of pancreatic tumours, expressions of other proteins involved in the process of angiogenesis were analysed in models of pancreatic cancer using a Proteome profiler angiogenesis array. Treatment of mice xenografted with human pancreatic cancer with *A platensis* led to an underexpression of endothelin 1, pentraxin 3 and acidic fibroblast growth factor and an overexpression of 3 angiogenic proteins in tumours, including VEGF‐A and AREG acting *via* VEGFR or EGFR (Table 1a). Using the same technique in an in vitro study on PA‐TU‐8902 cells, 26 overexpressed proteins were detected after a 2‐day exposure to *A platensis* extract (0.6 g/L) (Table 1b). Among them, VEGF‐A and AREG were also present, confirming our results from the xenograft tumours. We also detected EGF, an additional ligand of EGFR. As VEGF and EGF signalling pathways are inter‐related in pancreatic carcinogenesis,^23^ and AREG is an important prognostic factor of pancreatic cancer,^24^ we next focused on the possible role of the AREG pathway in the *A platensis*‐mediated therapeutic effects.

**Table 1:** The effect of *Arthrospira platensis* extract on the expression of angiogenic proteins in human PA‐TU‐8902 pancreatic cancer xenografts (a) and in vitro cultured cells (b)

Symbol	Definition	FDR	Fold change
(a) In vivo data^a^			
**VEGFA**	**Vascular endothelial growth factor A**	0.13	2.44
SERPINB5	Serpin peptidase inhibitor, clade B (ovalbumin), member 5	0.13	1.78
EDN1	Endothelin	0.13	0.44
PTX3	Pentraxin 3, long	0.13	0.54
**AREG**	**Amphiregulin**	0.24	1.53
FGF1	Fibroblast growth factor 1 (acidic)	0.24	0.67
(b) In vitro data^b^			
PLG	Plasminogen	0.001	124.82
SERPINF1	Serpin peptidase inhibitor, clade F (alpha‐2 antiplasmin, pigment epithelium‐derived factor), member 1	0.001	27.32
**AREG**	**Amphiregulin**	0.013	6.74
ARTN	Artemin	0.013	4.98
FGF7	Fibroblast growth factor 7	0.013	5.85
LEP	Leptin	0.013	5.52
THBS2	Thrombospondin 2	0.013	5.32
CSF2	Colony stimulating factor 2 (granulocyte‐macrophage)	0.017	3.65
ANGPT1	Angiopoietin 1	0.017	2.84
**EGF**	**Epidermal growth factor**	0.017	5.52
FGF4	Fibroblast growth factor 4	0.017	4.88
IL1B	Interleukin 1, beta	0.017	4.00
**VEGFA**	**vascular endothelial growth factor A**	0.017	7.47
ANGPT2	Angiopoietin 2	0.02	2.64
FGF2	Fibroblast growth factor 2 (basic)	0.024	3.74
ADAMTS1	ADAM metallopeptidase with thrombospondin type 1 motif, 1	0.025	42.03
ANG	Angiogenin, ribonuclease, RNase A family, 5	0.031	3.84
PRL	Prolactin	0.032	2.95
PTX3	Pentraxin 3, long	0.032	2.62
CCL3	Chemokine (C‐C motif) ligand 3	0.034	2.72
PROK1	Prokineticin 1	0.034	2.41
CCL2	Chemokine (C‐C motif) ligand 2	0.036	2.60
HGF	Hepatocyte growth factor (hepapoietin A; scatter factor)	0.037	1.92
TYMP	Thymidine phosphorylase	0.040	3.45
CXCL8	Chemokine (C‐X‐C motif) ligand 8	0.045	4.96
FGF1	Fibroblast growth factor 1 (acidic)	0.049	1.72

Note

Factors in bold were investigated in further detailed studies.

Abbreviation: FDR, false discovery rate.

a Using a Proteome profiler angiogenesis array covering 56 key angiogenic factors, 3 overexpressed proteins and 3 underexpressed (defined as the change of expression by at least 50%) were detected in tumour xenografts of mice treated orally with *A platensis* extract for 2 wk (0.5 g/kg once daily).

b Using the same array, 26 differentially expressed proteins (defined as FDR < 0.05) as a result of 2‐d exposure of human PA‐TU‐8902 pancreatic cancer cells to the *A platensis* extract (0.6 g/L) were detected.

### 3.6 The role of EGF/AREG in *A platensis*‐mediated therapeutic effects

**Figure 4:**
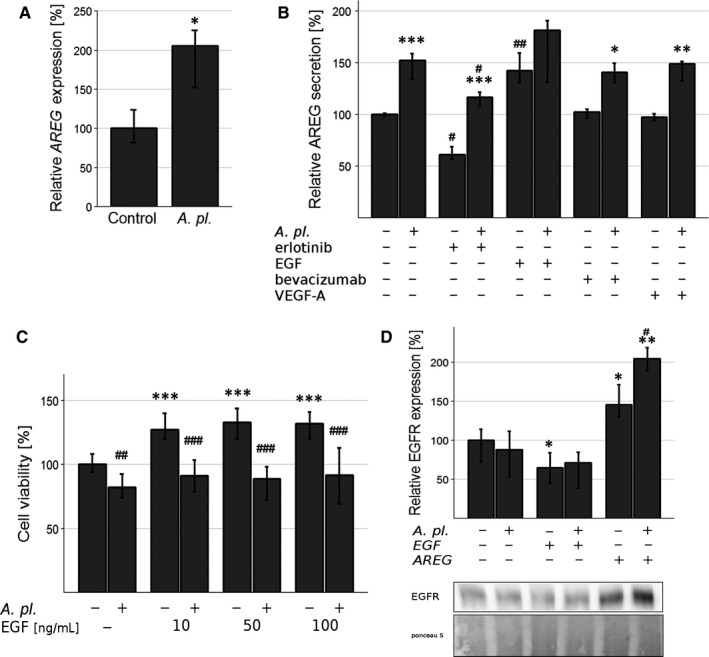
The role of EGF/AREG in *Arthrospira platensis* ‐mediated therapeutic effects in human PA‐TU‐8902 pancreatic cancer cells. A, AREG *mRNA e*xpression after 24‐h exposure to *A platensis* extract. *P < .05 vs control; B, AREG protein secretion after 24‐h exposure to *A platensis* extract and/or other angiogenesis modulating factors. bevacizumab (25 μg/L), erlotinib (1 μmol/L), rVEGF‐A, rEGF (both 50 μg/L). *P < .05, **P < .01, ***P < .001 control vs* A platensis*; ^#^ *P* < .05, ^##^ *P* < .01 group vs group + erlotinib/rEGF/bevacizumab/rVEGF‐A; C, The effect of 24‐h exposure of *A platensis* extract and/or 10‐min stimulation by rEGF and rAREG (both 50 μg/L) on EGFR protein expression. *P < .05; **P < .01 group vs group + rEGF/rAREG; ^#^ *P* < .05, control vs *A platensis* (D) The effect of *A platensis* extract and exogenous rEGF (50 μg/L) on pancreatic cancer cell proliferation. ***P < .001 group vs group + rEGF; ^##^ *P* < .01, ^###^ *P* < .001 control vs *A platensis*. Data expressed as % of control values and represent median ± IQ range. *A pl.*, *A platensis*

The expression of mRNA of AREG (an EGFR ligand) in PA‐TU‐8902 cells treated with *A platensis* extract was up‐regulated to 205% (Figure 4A, *P* < .05). In line with this result, increased production of the AREG protein in treated pancreatic cancer cells was also observed, and this trend was consistent even when *A platensis* extract was co‐administered with the majority of other angiogenesis modulating compounds (Figure 4B). Recombinant VEGF‐A or bevacizumab did not influence the level of AREG in the control cells; however, a significant increase in AREG was observed in *A platensis*‐treated cells. On the other hand, erlotinib (an EGFR inhibitor) decreased the AREG level in both the control cells (by 41%, *P* < .05) and the *A platensis*‐treated cells (by 36%, *P* < .05). Simultaneously, recombinant EGF markedly increased the AREG level in the control cells (to 142%, *P* < .01); and an additional exposure to *A platensis* further increased its production (Figure 4B). Interestingly, no EGF was detected in the medium of control cells or in cells treated with *A platensis* extract.

In contrast to both VEGF‐A and AREG having no effect, recombinant EGF added to the culture medium consistently increased proliferation of PA‐TU‐8902 cells (Figure 4C). Interestingly, treatment with *A platensis* extract (0.3 g/L) eliminated this effect of EGF (Figure 4C). Exposure of PA‐TU‐8902 cells to exogenous EGF (50 mg/L) did not have any effect on either VEGF‐A mRNA or protein expression.

*A platensis* extract did not affect EGFR protein expression in PA‐TU‐8902 cells after 24 hours (Figure 4D). In contrast, EGF treatment led to down‐regulation of EGFR protein expression, whereas AREG had the opposite effect (this effect was co‐stimulated by *A platensis* extract, Figure 4D).

### 3.7 *Arthrospira platensis* interferes with ERK activation and potentiates AKT activation

**Figure 5:**
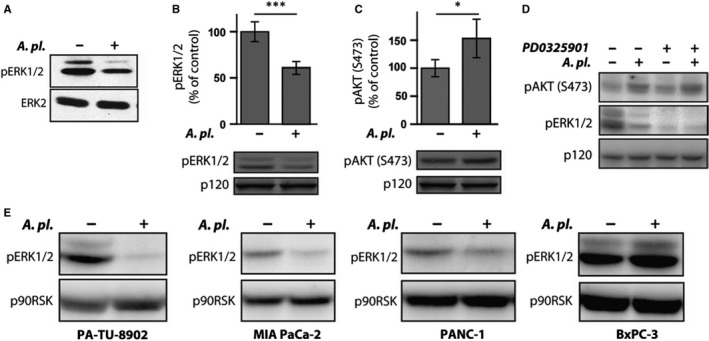
The effect of *Arthrospira platensis* extract on ERK and AKT activity in human PA‐TU‐8902 pancreatic cancer cells. A, *A platensis* suppresses ERK activation in PA‐TU‐8902 cells. Cell lysates from cells treated with *A platensis* extract for 1 h were probed with antibody recognizing active, doubly phosphorylated ERK (pERK1/2) to determine the activation level of ERK. In parallel, cell lysate blots were re‐probed with an ERK2 antibody to confirm equal protein loading. B, Quantification of ERK activity in *A platensis*‐treated PA‐TU‐8902 cells. Cell lysates from (A) run in duplicates were probed with antibody recognizing active, doubly phosphorylated ERK (pERK1/2) and IR700 dye‐labelled secondary antibody. The fluorescent signal was normalized to p120RasGap probed with IR800 dye secondary antibody. Data are plotted as mean ± SD, with the ERK activity in untreated cells as a reference. C, Quantification of AKT activity in *A platensis*‐treated PA‐TU‐8902 cells. Cell lysates from (A) run in duplicates were probed with antibody recognizing active AKT (pAKT‐S473) and IR700 dye‐labelled secondary antibody. The fluorescent signal was normalized to p120RasGap probed with IR800 dye secondary antibody. Data are plotted as mean ± SD, with the AKT activity in untreated cells as a reference. D, Effect of ERK inhibition on the phosphorylation of AKT in PA‐TU‐8902 cells. Cell lysates from cells treated with MEK inhibitor PD0325901 (1 μmol/L) and *A platensis* extract for 1 h were probed with antibody recognizing phosphorylated ERK (pERK1/2) and phosphorylated AKT (pAKT‐S473) to determine the activation level of ERK and AKT, respectively, and HRP‐labelled secondary antibody. Equal protein loading was confirmed by blotting for p90RSK. E, *A platensis* inhibits ERK activation in different pancreatic cancer cell lines. Cell lysates from pancreatic cancer cell lines (PA‐TU‐8902, MIA PaCa‐2, PANC‐1 and BxPC‐3) treated with *A platensis* extract for 1 h were probed with antibody recognizing active, doubly phosphorylated ERK (pERK1/2) and HRP‐labelled secondary antibody to determine the activation level of ERK. Equal protein loading was confirmed by blotting for p90RSK

In pancreatic cancers, *KRAS* is almost invariably mutated, where it activates downstream ERK and PI3K/AKT signalling pathways to promote proliferation, survival and invasion of cancer cells.^25^, ^26^ Due to the key roles of the ERK signalling pathway in tumour cell progression, the effects of *A platensis* on ERK phosphorylation were also analysed in PA‐TU‐8902 cells that harbour Ras (G12V) activating mutation. One‐hour exposure of PA‐TU‐8902 pancreatic cancer cells to *A platensis* extract led to a significant decrease in ERK phosphorylation (Figure 5A,B). Similar effect of *A platensis* extract on ERK inhibition was also observed in PANC‐1 and MiaPaCa‐2 pancreatic cancer cell lines, while no inhibitory effect was seen in BxPC‐3 cells (Figure 5E). Surprisingly, exposure of PA‐TU‐8902 pancreatic cancer cells to *A platensis* extract led to a slight but reproducible increase in phosphorylation of AKT (Figure 5C). The up‐regulation of PI3K activity and AKT phosphorylation could be a consequence of the inhibition of the ERK pathway.^27^ We thus examined whether pharmacological inhibition of the ERK pathway activates AKT in PA‐TU‐8902 cells. Indeed, we found that the cells treated with MEK inhibitor PD0325901 increased the phosphorylation of AKT (Figure 5D). These data indicate that *A platensis* extract induces AKT phosphorylation at least partly through the negative crosstalk with the ERK pathway and possibly also independently of ERK by so far unknown mechanism.

### 3.8 *Arthrospira platensis* inhibits ERK activity upstream of Raf and affects the expression of ERK‐regulated proteins

**Figure 6:**
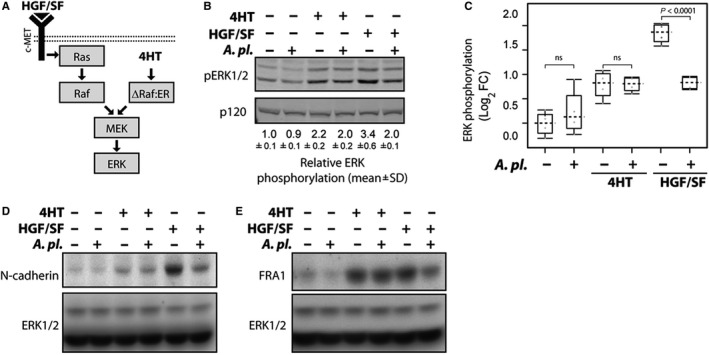
The effect of *Arthrospira platensis* extract on ERK pathway activation and the expression of N‐cadherin and FRA1 in ΔRaf‐1:ER MDCK cells. A, Schematic representation of ERK activation in response to growth factor HGF/SF or by activation of ΔRaf‐1:ER by 4HT in MDCK cells. HGF/SF (HGF/SF is indicated by a black triangle) and c‐Met signals through Ras to activate Raf‐Mek‐ERK signalling cascade. The right‐hand part illustrates the Ras‐independent, direct activation of the ERK pathway by conditionally active ΔRaf‐1:ER. ΔRaf‐1:ER contains constitutively active domain of Raf‐1 fused to the hormone binding domain of the estrogen receptor (ER). ER holds Raf‐1 protein in the inactive conformation. 4‐hydroxytamoxifen (4HT) induces rapid ΔRaf‐1:ER conversion to active state followed by sustained ERK activation.^19^ B, *A platensis* suppresses Ras‐dependent ERK activation. MDCK ΔRaf‐1:ER cells were stimulated with either 4HT (1 μmol/L) or HGF/SF (100 ng/mL) for 8 h. Cell lysates were probed with antibodies recognizing active phosphorylated ERK (pERK1/2) and p120RasGAP. Relative ERK phosphorylation (pERK1/2 signal normalized to p120RasGAP expression from experiment performed in duplicates) is shown below the blot (mean ± SD), with ERK activity in untreated cells as a reference. C, *A platensis* inhibits ERK phosphorylation in cells stimulated with HGF/SF. Box‐and‐whisker plot showing Log_2_ fold change in ERK phosphorylation in response to HGF/SF and 4HT. Dashed line indicates median and grey circles individual data point. ns—not significant. D, E, *A platensis* suppresses the expressions of ERK‐regulated proteins N‐cadherin and FRA1. MDCK ΔRaf‐1:ER cells were stimulated with either 4HT or HGF/SF for 24 h, and lysates were probed with N‐cadherin (panel d) and FRA1 (panel e) antibodies. Equal protein loading was confirmed by blotting for ERK1/2

ERK activation is in direct association with high malignant potential of pancreatic cancer cell invasion of cancer cells.^25^ To gain further insight into the molecular mechanisms of an inhibitory function of *A platensis*, we examined whether *A platensis* was able to modulate this signalling system in response to different stimuli. We utilized epithelial MDCK cells stably expressing a conditional active version of Raf (ΔRaf‐1:ER).^19^ In these cells, sustained ERK activity can be induced by two different means. ERK can be activated by extracellular HGF/SF via its cognate receptor, c‐Met (c‐mesenchymal‐epithelial transition factor). In fact, the HGF/Met signalling pathway is importantly involved in pathogenesis of pancreatic cancer, and HGF/Met inhibitors are believed to become in near future crucial therapeutics for pancreatic cancer management.^28^ Importantly, HGF/SF then activates the signalling components, including Ras, that are required for the signal transduction from the c‐Met receptor towards the ERK pathway (Figure 6A). ERK can also be activated more directly by 4HT, which converts ΔRaf‐1:ER protein to an active form, and then subsequently activates the ERK pathway in a Ras‐independent manner (Figure 6A). Although this model is not related to pancreatic cancer, examining the effect of *A platensis* on HGF/c‐Met‐Ras and Raf‐mediated ERK activation could help to assess the mechanisms of *A platensis* impact on the ERK pathway.

When the cells were treated with HGF/SF, *A platensis* inhibited both acute and sustained ERK activation (Figure 6B and Figure S1). In contrast, little or no *A platensis*‐mediated inhibition of ERK activity was seen in the initial experiments, where ERK activation was induced by ΔRaf‐1:ER (Figure 5D,E). This finding raised the possibility that *A platensis* functions upstream of Raf. To further explore this concept, we performed side‐by‐side comparisons of *A platensis* function in cells stimulated with either HGF/SF or 4HT *A. platensis* inhibited ERK phosphorylation in cells stimulated with HGF/SF, but had no significant effect on ERK activation in cells stimulated with 4HT (Figure 6C).

We also examined whether *A platensis* treatment compromises the expression of proteins regulated by ERK. ERK activity in MDCK cells is necessary and sufficient for the expression of several pro‐migratory proteins including N‐cadherin and the transcription factor FRA1.^29^ *A platensis* extract substantially inhibited both N‐cadherin and FRA1 expression induced by HGF/SF, however, not in 4HT‐treated cells (Figure 6D,E). Taken together, these data suggest that *A platensis* inhibits ERK activation upstream of Raf at the level of Ras and subsequently suppresses the expression of ERK‐regulated proteins.

## 4 DISCUSSION

Despite the medical progress achieved over the last few decades, cancer still poses a major threat to worldwide public health, with increasing incidence rates in most countries.^30^ Diet is among the most important factors affecting the risks of cancer.^31^ Besides the role of the composition of macro‐nutrients, specific food components have been shown to contribute substantially to the prevention of carcinogenesis, modulating various stages in this process including apoptosis, angiogenesis and metastasis.^32^ In fact, angiogenesis plays a crucial role in tumour progression, making it an attractive target for novel cancer therapies.^12^

In our study, we have explored the potential anti‐angiogenic activities of an extract of *A platensis*, a freshwater blue‐green alga commonly used as a nutraceutical.^33^ Indeed, the apparent antiproliferative effects of *A platensis* seen in our previous studies^4^ might at least partially be due to the lowered vascularization of the pancreatic tumours, as evidenced by decreased expression of CD31, together with a lowered invasion potential of the treated cells. Surprisingly, this phenomenon was accompanied by an increased production of VEGF‐A and other angiogenic factors. Tumour angiogenesis is a complex process, which involves a highly regulated orchestration of multiple signalling pathways, and whose impairment certainly evokes an array of feedback mechanisms, as can clearly be documented by the differential expressions of the multiple angiogenic factors seen in our study.

The relationship between CD31 in tumours and VEGF‐A expressions is not obvious. Although a positive (and expected) association was reported in one study on patients with pituitary adenomas,^21^ this has not been confirmed by others in the same tumours,^34^ nor in osteosarcoma^35^ and/or in breast cancer.^36^, ^37^ On the other hand, VEGF‐A overexpression is a potent prognostic marker in patients with pancreatic cancer, indicating increased malignancy of the tumours^38^; further, the same association as with the prognosis of pancreatic cancer has also been reported for AREG.^24^ We have shown proof of an increased production of VEGF‐A in several independent experiments, both in vivo and in vitro, as well as at both the mRNA and the protein level. The same up‐regulation was also observed for other angiogenic factors, including AREG acting as a ligand for EGFR. This seems to be important, as the EGFR pathway plays an important role in Ras‐mutated pancreatic cancers, with direct impacts on the ERK and AKT signalling pathways.^18^

One could logically speculate that the overexpression of VEGF‐A observed in our study was due to a positive feedback mechanism caused by VEGFR inhibition. In fact, an untouched VEGF‐A production was observed with vatalanib, a selective VEGFR inhibitor, in both experimental and clinical studies on various cancers including pancreatic carcinoma.^39^, ^40^, ^41^ In our study, vatalanib treatment of pancreatic cancer cells resulted in exactly the same effect on VEGF‐A overproduction as exposure to an *A platensis* extract did. However, the expression of *VEGF‐A* receptors 1 and 2 in our pancreatic cancer cells was not affected by the *A platensis* treatment, indicating that the mechanism of *A platensis* extract‐induced expression of VEGF‐A must be mediated *via* some other means.

Our results on the increased production of VEGF‐A, induced by *A platensis* extract, seem to be in line with those of Loboda et al who demonstrated that administration of biliverdin (a potent anticancer and also anti‐angiogenic tetrapyrrolic compound^42^) remarkably increased VEGF‐A expression in keratinocytes.^43^ These data suggest that VEGF‐A could be up‐regulated by related tetrapyrrolic compounds, such as phycocyanobilin present in *A platensis*.^4^

It is also important to note that intratumour VEGF‐A levels negatively correlate with the invasiveness of glioblastoma multiforme tumour cells^44^; in fact, VEGF‐A was reported as a negative regulator of receptor tyrosine kinases, by blocking their activation through direct interaction.^44^ These facts may account for tumour relapses during anti‐VEGF‐A therapy 44 and suggest the heterogeneity of the biological action of VEGF‐A in carcinogenesis. Importantly, VEGF‐A production is also under the control of EGFR ligands, as has been reported for AREG in human chondrosarcoma cells.^45^ Notably, AREG was also up‐regulated in our *A platensis*‐treated pancreatic cancer cells.

One of the anticancer mechanisms of *A platensis* treatment seems to be inhibition of the ERK signalling pathway. The aberrant activation of the ERK signalling pathway, composed of the Raf, MEK and ERK protein kinases, is directly associated with the high malignancy potential of numerous cancers, including pancreatic ductal carcinomas.^25^ The ERK pathway is prototypically activated by small GTPase Ras, which directly associates with RAF, and promotes its activation.^46^ Ras mutational activation is also the main oncogenic signalling pathway in pancreatic cancer,^47^ and the PA‐TU‐8902 pancreatic cancer cells used in our study also contain the Ras(G12V) oncogenic mutation.^48^ Our results from the MDCK cell line expressing conditionally active Raf deletion mutant show that *A platensis* inhibits ERK activation by Ras(G12V), but not Raf. In addition, we found that *A platensis* extract does not affect ERK activity in BxPC3 pancreatic cancer cells that contain constitutively active B‐RAF due to in‐frame deletion.^49^ As active Ras promotes Raf activation by direct association, these data indicate that *A platensis* most likely inhibits the ERK pathway activation at the level or upstream of Ras or that it affects Ras‐mediated activation of Raf.

Activation of the ERK pathway plays a central role in cell proliferation, as activated ERK phosphorylates several transcription factors involved in cell cycle entry and cell proliferation.^14^ Additionally, the activation of ERK promotes cell migration and invasion.^19^, ^29^ Consistent with this pleiotropic function of ERK, we found that inhibition of ERK by *A platensis* correlates with impaired cell proliferation, migration and invasion. ERK promotes cell migration on several levels, as it can increase the rate of focal adhesion turnover,^50^, ^51^ and also increase the expression of pro‐migratory genes by activating transcription factor FRA1.^29^ Finally, ERK can promote epithelial cell migration, and it can induce disruption of cell‐cell contacts by an epithelial‐mesenchymal transition‐like process.^52^ Our findings from MDCK cells show that upon HGF/SF stimulation, *A platensis* suppresses the expression of both the transcription factor FRA1 and the epithelial‐mesenchymal transition marker N‐cadherin and further support the hypothesis that *A platensis* inhibits pro‐malignancy ERK signalling. HGF/SF signalling has an important function in pancreatic cancer pathology. c‐Met, the receptor for HGF/SF, is expressed on pancreatic cancer cells, and HGF/SF is produced by pancreatic stellate cells in tumour environment.^53^ Such paracrine signalling plays a role in pancreatic cancer cell proliferation and invasion. In addition, HGF/c‐Met signalling may potentiate the production of angiogenic factors^54^, ^55^ raising the possibility that HGF/c‐Met signalling induces angiogenic factors also in pancreatic cancer.

The elevated expression of VEGF‐A and amphiregulin, together with inhibition of the mitogenic RAF‐MEK‐ERK signalling cascades seen upon treatment with *A platensis* extract, was surprising, as ERK signalling is generally believed to induce VEGF‐A production.^56^ We speculate that there are pathways parallel to ERK that promote VEGF‐A expression. The primary candidate for the pathway acting parallel to ERK is the PI3K‐AKT pathway.^57^ In accord with our results, it has been shown that the inhibition of the ERK pathway resulted in up‐regulation of PI3K activity and AKT phosphorylation in different cell lines including breast and prostate cancer.^58^, ^59^ Concordantly, ERK can impose a negative crosstalk towards PI3K‐AKT by phosphorylation of GAB1 adaptor protein and decoupling PI3K from growth factor receptors,^60^ by phosphorylation of upstream receptor^61^ or by inducing PTEN translocation to the membrane and to the close proximity of PI3K*.* 62 Interestingly, the relief from the negative crosstalk and the PI3K activation depends on the mutational status of Ras or Raf proteins. The MEK inhibition in cells with wild‐type and mutated Ras/Braf resulted in inhibition and activation of PI3K‐AKT axis, respectively.^27^ Ras mutational activation is the main oncogenic signalling pathway in pancreatic cancer,^47^ and human PA‐TU‐8902 pancreatic cancer cells used in our study also contain Ras(G12V) oncogenic mutation*.* 48 Taken together, these data indicate that in PA‐TU‐8902 pancreatic cell line *A platensis* extract can partially disable a negative feedback imposed on PI3K‐AKT by constitutively activated Ras‐ERK pathway. Increased PI3K/AKT signalling and probably additional pathways parallel to ERK then promote VEGF‐A expression.

In conclusion, we have confirmed the inhibitory effects of an *A platensis* extract on PA‐TU‐8902 pancreatic cancer cells and have identified its angiogenic mechanisms to be the important targets of these activities. Surprisingly, these effects were accompanied by increased production of VEGF and other angiogenic factors, presumably as a result of feedback deregulation of the angiogenic process of the treated tumour cells. Further detailed studies on multiple pancreatic cancer cells as well as tumour microenvironment and immune system factors are needed to verify whether observed anti‐angiogenic activities an *A platensis* extract are a general phenomenon.

## CONFLICT OF INTEREST

The authors declare no conflict of interests.

## AUTHOR CONTRIBUTIONS

IM performed cell culture studies, angiogenesis and proteome profiler assays. RK performed cell culture studies. KV and ML performed wound‐healing and tube‐like formation assays. ML, MH, HS, MK and JŠ carried out qPCR assays and statistical analyses. JR, ZK and TV performed ERK/AKT analyses. TV designed the molecular mechanism studies. IN and PN carried out immunohistochemistry and WB analyses. LV designed the whole study, planned the experiments and interpreted the data. All authors contributed to writing and reading of the final manuscript.

## Data Availability

All data generated or analysed during this study are included in this published article (and its supplementary information files).
