# Supplementary material for: Anti‐angiogenic effects of the blue‐green alga Arthrospira platensis on pancreatic cancer
Source: J Cell Mol Med. 2020 Jan 19;24(4):2402–15. doi: 10.1111/jcmm.14922 (PMC7028863; doi:10.1111/jcmm.14922)
Supplement: Supplementary file 1 [file JCMM-24-2402-s001.docx]

**Suppl. Tab.** **1. Antibodies used for western blot analyses.**

| Primary antibody | Secondary antibody |
| --- | --- |
| rabbit anti-VEGF (Abcam, Cambridge, UK; 1:1,000) | HRP - F(ab’)2 fragment donkey anti-rabbit antibody (GE Healthcare, Chicago, IL, USA; 1:2,000) |
| goat anti-CD31 (Santa Cruz Biotechnology, CA, USA; 1:100) | HRP - rabbit anti-goat immunoglobulin G (Pierce Biotechnology, Rockford, IL, USA;1:5,000) |
| mouse anti-β-actin (Sigma-Aldrich; 1:5,000) |  |
| rabbit anti-EGFR XP^®^ (Cell Signaling Technology, Danvers, MA, USA; 1:1,500) | HRP - anti-rabbit antibody (Dako, Glostrup, Denmark; 1:3,400) |
| rabbit anti-phospho-p44/42 MAPK (Cell Signaling Technology, Danvers, MA, USA; 1:1,000) rabbit anti-phospho-Akt (Ser473) (Cell Signaling Technology, Danvers, MA, USA; 1:1,000) | anti-rabbit IRDye 700 (LI-COR Biosciences, Lincoln, NE, USA; 1:10,000) Goat Anti-Rabbit IgG-HRP (Sigma-Aldrich (St. Louis, MO, USA); 1:5,000) |
| FRA1 (R20) (Santa Cruz Biotechnology, CA, USA; 1:1,500) |  |
| mouse anti-p44/42 MAPK (Erk1/2) (Cell Signaling Technology, Danvers, MA, USA; 1:1,000) | anti-mouse IRDye 800 (LI-COR Biosciences, Lincoln, NE, USA; 1:10,000)Goat Anti-Mouse IgG-HRP (Sigma-Aldrich (St. Louis, MO, USA); 1:10,000) |
| mouse anti-p120RasGAP (ECM Biosciences LLC, Versailles, KY, USA; 1:1,000)p90RSK (BD Biosciences, San Jose, CA, USA; 1:2,500)N-cadherin (BD Biosciences, San Jose, CA, USA; 1:1,000)ERK2 (clone B3B9, gift from M.J. Weber 1: 5,000) |  |

**Suppl. Tab. 2. Primer sequences for target and reference genes.**

| *Gene* | Forward primer | Reverse primer | Product size (bp) |
| --- | --- | --- | --- |
| *AREG* | TCGGGAGCCGACTATGACTAC | TTCACTTTCCGTCTTGTTTTGG | 126 |
| *EGF* | ATAAGCGGCTGTTTTGGATTC | GGTCACCAAAAAGGGACATTG | 141 |
| *EGF* | TTCTGTCCTGAAGGCTCAGTG | CCAGGATACTGGGCTAAGAGG | 114 |
| *EGFR* | TAACTGTGAGGTGGTCCTTGG | GAGGGCAATGAGGACATAACC | 112 |
| *HBEGF* | AGAAGAGGGACCCATGTCTTC | CAGCCCATGACACCTCTCTC | 131 |
| *HPRT* | CACTGGCAAAACAATGCAGAC | GGGTCCTTTTCACCAGCAAG | 92 |
| *VEGFA* | ACTGAGGAGTCCAACATCACC | CTGCATTCACATTTGTTGTGC | 104 |
| *VEGFR1* | TGGTTTGCT TGAGCTGTGTTC | TTTGCCTGAAATGGTGAGTAAGG | 117 |
| *VEGFR2* | TGCTTCTACCGGGAAACTGAC | ACACGACTCCATGTTGGTCAC | 103 |

*AREG*, amphiregulin; EGF, epidermal growth factor; EGFR, epidermal growth factor receptor; HBEGF, heparin binding epidermal growth factor; *HPRT,* hypoxanthine-guanine phosphoribosyl transferase; *VEGFA*, vascular endothelial growth factor A; *VEGFR1/2*, vascular endothelial growth factor receptor 1/2.

**Suppl. Figures**

**Suppl. Fig. 1. ERK by *A. platensis* inhibits both acute and sustained ERK - induced by activation by HGF/SF, but not by 4HT.**

**
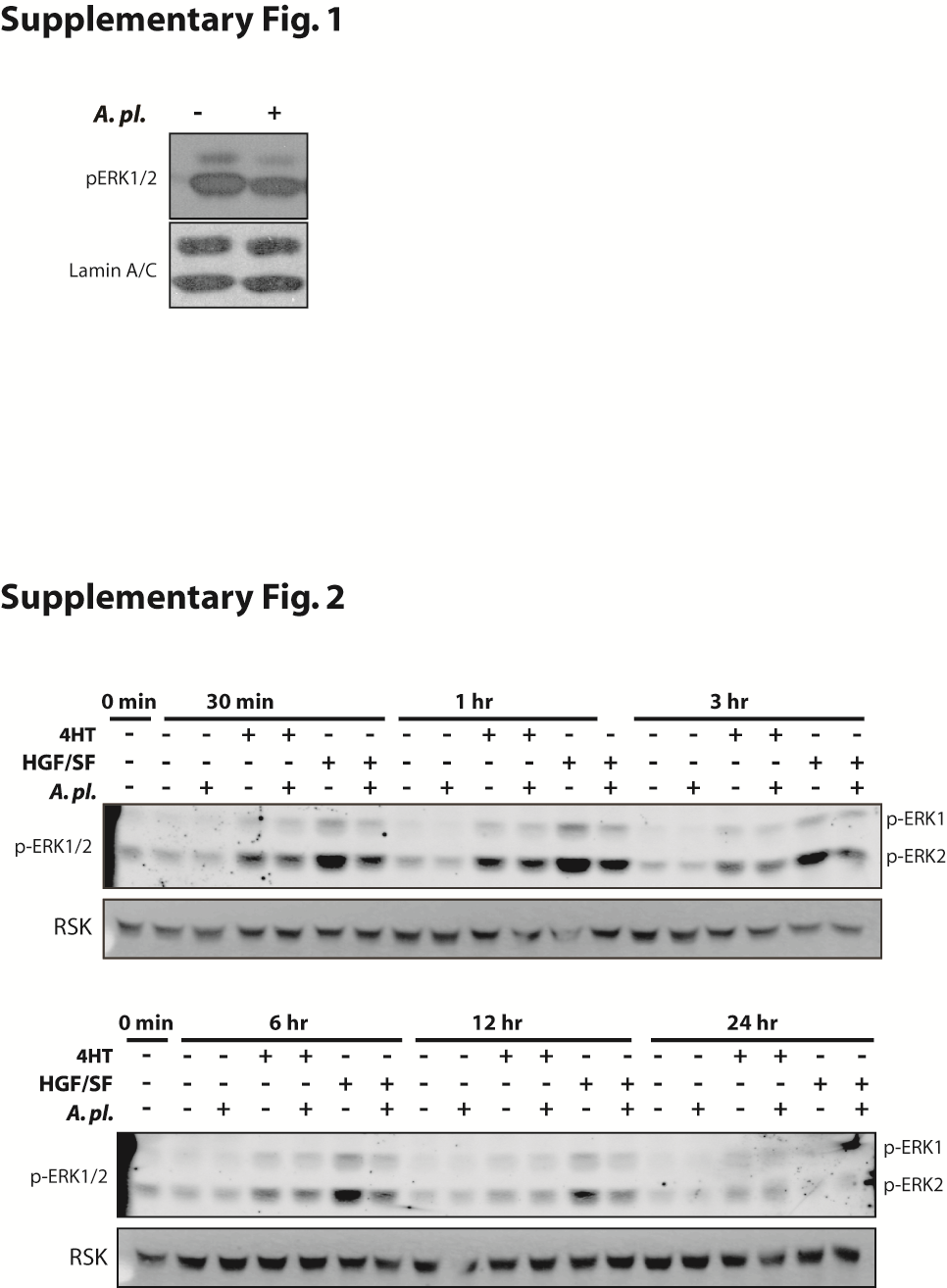
**

MDCK cells constitutively expressing ΔRaf-1:ER (constitutively active C-terminal portion of Raf-1 fused to hormone binding domain of the estrogen receptor) were pretreated with *A. platensis* extract (0.3 g/L) for 60 min, and challenged for the indicated times with either HGF/SF or 4HT. ERK activation was determined by probing cell lysates by antibody recognizing active, doubly phosphorylated ERK, and equal protein loading was confirmed by blotting for RSK.
